# Expression Quantitative Trait Loci in Equine Skeletal Muscle Reveals Heritable Variation in Metabolism and the Training Responsive Transcriptome

**DOI:** 10.3389/fgene.2019.01215

**Published:** 2019-11-26

**Authors:** Gabriella Farries, Kenneth Bryan, Charlotte L. McGivney, Paul A. McGettigan, Katie F. Gough, John A. Browne, David E. MacHugh, Lisa Michelle Katz, Emmeline W. Hill

**Affiliations:** ^1^UCD School of Agriculture and Food Science, University College Dublin, Dublin, Ireland; ^2^UCD School of Veterinary Medicine, University College Dublin, Dublin, Ireland; ^3^UCD Conway Institute of Biomolecular and Biomedical Research, University College Dublin, Dublin, Ireland; ^4^Research and Development, Plusvital Ltd., Dublin, Ireland

**Keywords:** expression quantitative trait loci, gene expression, RNA sequencing, horse, exercise, aerobic metabolism

## Abstract

While over ten thousand genetic loci have been associated with phenotypic traits and inherited diseases in genome-wide association studies, in most cases only a relatively small proportion of the trait heritability is explained and biological mechanisms underpinning these traits have not been clearly identified. Expression quantitative trait loci (eQTL) are subsets of genomic loci shown experimentally to influence gene expression. Since gene expression is one of the primary determinants of phenotype, the identification of eQTL may reveal biologically relevant loci and provide functional links between genomic variants, gene expression and ultimately phenotype. Skeletal muscle (gluteus medius) gene expression was quantified by RNA-seq for 111 Thoroughbreds (47 male, 64 female) in race training at a single training establishment sampled at two time-points: at rest (*n* = 92) and four hours after high-intensity exercise (*n* = 77); *n* = 60 were sampled at both time points. Genotypes were generated from the Illumina Equine SNP70 BeadChip. Applying a False Discovery Rate (FDR) corrected *P*-value threshold (*P*_FDR_ < 0.05), association tests identified 3,583 *cis*-eQTL associated with expression of 1,456 genes at rest; 4,992 *cis*-eQTL associated with the expression of 1,922 genes post-exercise; 1,703 *trans*-eQTL associated with 563 genes at rest; and 1,219 *trans*-eQTL associated with 425 genes post-exercise. The gene with the highest *cis*-eQTL association at both time-points was the endosome-associated-trafficking regulator 1 gene (*ENTR1*; Rest: *P*_FDR_ = 3.81 × 10^-27^, Post-exercise: *P*_FDR_ = 1.66 × 10^-24^), which has a potential role in the transcriptional regulation of the solute carrier family 2 member 1 glucose transporter protein (SLC2A1). Functional analysis of genes with significant eQTL revealed significant enrichment for cofactor metabolic processes. These results suggest heritable variation in genomic elements such as regulatory sequences (e.g. gene promoters, enhancers, silencers), microRNA and transcription factor genes, which are associated with metabolic function and may have roles in determining end-point muscle and athletic performance phenotypes in Thoroughbred horses. The incorporation of the eQTL identified with genome and transcriptome-wide association may reveal useful biological links between genetic variants and their impact on traits of interest, such as elite racing performance and adaptation to training.

## Introduction

In the 6,000 years since horses were first domesticated on the Eurasian steppe, there has been strong artificial selection for various athletic traits (Levine, 1999). Selection for athleticism is perhaps most clearly manifested in the Thoroughbred, which has undergone over 300 years of intense selection for speed and racing performance (Willett, 1975; Todd et al., 2018). As a result the Thoroughbred has a highly developed musculature, with a skeletal muscle mass ∼10% greater than other horse breeds (∼55% compared to ∼42%) (Gunn, 1987), accompanied by decreased body fat (Kearns et al., 2002), superior glycogen storage capacity (Votion et al., 2012), increased mitochondrial volume (compared to other mammals) (Kayar et al., 1989) and a high degree of plasticity in skeletal muscle fibre composition (Rivero, 2004).

The response of equine skeletal muscle to training has been well studied (Snow et al., 1985; Snow, 1994). These responses in general increase the oxidative capacity of the muscle, such as fibre type switching from fast-twitch glycolytic fibres to slow-twitch, high-oxidative fibres (Snow, 1994; Serrano et al., 2000), an increase in oxidative phosphorylation (Snow et al., 1985; Votion et al., 2012) and increased mitochondrial volume (Tyler et al., 1998). Training also elicits an increase in skeletal muscle mass (Rivero et al., 1996), mediated through hyperplastic growth as opposed to marked hypertrophy (Rivero et al., 1996; Rivero et al., 2002).

The transcriptional response to exercise and training in skeletal muscle has been studied in the Thoroughbred (McGivney et al., 2009; Eivers et al., 2010; McGivney et al., 2010; Eivers et al., 2012; Bryan et al., 2017). Initially, reverse transcription quantitative real-time polymerase chain reaction (RT-qPCR) was used to quantify expression of 18 candidate genes in response to a standardised exercise test on a high-speed treadmill (Eivers et al., 2010). Significant differential expression of creatine kinase M-type (*CKM*), cytochrome c oxidase subunit 4I1 (*COX4I1*), cytochrome c subunit 4I2 (*COX4I2*), pyruvate dehydrogenase kinase 4 (*PDK4*), PPARG coactivator 1 alpha (*PPARG1A*) and solute carrier family 24 member 4 (*SLC2A4*) four hours post-exercise was detected. *PPARG1A* is a transcription factor downstream of hypoxia-inducible factor (HIF), activation of *PPARG1A via* HIF in response to exercise induces downstream adaptations in oxidative phosphorylation (Arany, 2008). The differentially expressed genes were downstream targets of HIF or related to oxidative phosphorylation or muscle substrate use (Kraniou et al., 2006) The availability of a dedicated equine microarray allowed gene expression to be measured across 9,333 expressed sequence tags (ESTs). This technology was then used to examine the changes in gene expression induced by exercise, without *a priori* knowledge of the genes involved (McGivney et al., 2009). Analysis of the differentially expressed genes showed a functional enrichment of genes involved in insulin signalling, focal adhesion, hypertrophic and apoptotic pathways. Digital gene expression was used to investigate the transcriptional response to a ten-month training protocol (McGivney et al., 2010), identifying functional enrichment of genes relevant to aerobic metabolism. More recently, RNA sequencing (RNA-seq) was used to investigate the response to both exercise and training, and a network biology approach was employed to identify relevant functional modules that highlighted the role of autophagy (Bryan et al., 2017) While these studies provide insight to the genes involved in the transcriptional response to exercise, they do not reveal whether there is variation in the transcriptional response among individuals and how this may influence skeletal muscle function.

Expression quantitative trait loci (eQTL) are genomic variants, typically single nucleotide polymorphism (SNPs), that are associated with variation in RNA transcript abundance. Jansen and Nap (2001) introduced the concept of ‘genetical genomics’ where genomic loci were associated with cellular intermediates, such as transcript abundance, to catalogue functional relevance for non-coding variants. These measurements at a cellular level then act as endophenotypes, which are heritable, intermediate phenotypes This was a particularly important development because the clear majority (>85%) of QTLs detected in genome-wide association studies (GWAS) are located in non-coding regions (Hindorff et al., 2009; Brown et al., 2013).

*Cis*-eQTL are genetic variants that alter gene expression in an allele-specific manner and are typically located in gene regulatory regions (Wittkopp, 2005; Westra and Franke, 2014). Identification of true *cis*-eQTL requires aligning reads to their chromosome of origin; consequently, many studies have by convention defined any eQTLs within 1 Mb of the transcription start site (TSS) of the gene they act on as *cis*. Conversely, *trans*-eQTL act in a less direct manner, altering the expression of a secondary genome product—for example, a transcription factor or a microRNA—that regulates expression of a distant gene elsewhere in the genome (Wittkopp, 2005).

The study of eQTL in skeletal muscle to-date has been largely to investigate functional variants in the pathogenesis of type II diabetes (T2D) in humans (Mason et al., 2011; Keildson et al., 2014b; Sajuthi et al., 2016). While GWAS for T2D have identified loci associated with disease risk, these studies have not provided information on the function of these variants or the mechanism by which they contribute to disease. Keildson et al. (2014b) performed an eQTL investigation using skeletal muscle biopsies from 104 human subjects and identified an association between the rs4547172 SNP and muscle phosphofructokinase gene (*PFKM*) expression. Furthermore, the study found that increased expression of *PFKM* was associated with increased resting plasma insulin (an endophenotype) and T2D (an end-point phenotype). This example shows that an eQTL approach can identify functional links between genomic variants, gene expression, endophenotypes, and ultimately, disease.

Variation in human gene expression has been found to be highly heritable (Monks et al., 2004; Stranger et al., 2007; Wright et al., 2014). Given the influence of gene expression on phenotype, detection of heritable variation in skeletal muscle gene expression may provide insight into genomic loci contributing to variation in exercise and performance related phenotypes.

In this study, we hypothesised that there is heritable variation in the Thoroughbred skeletal muscle transcriptional response to exercise and training, and that this variation may have implications for athletic performance.

## Methods

### Ethics Statement

University College Dublin Animal Research Ethics Committee approval (AREC-P-12-55-Hill), a licence from the Department of Health (B100/3525), and informed owner consent were obtained.

### Cohort

Skeletal muscle biopsy samples (gluteus medius) were collected from 111 horses (47 male, 64 female) born between 2011 and 2012. All horses were based at a single training yard, under the supervision of a single trainer and under similar management and feeding regimes. The 111 horses used for the study were produced from 19 different sires and 94 different dams.

Biopsies were collected at two time points: untrained at rest (UR) and untrained four hours post-exercise (UE). Of the 111 horses, 60 were sampled at both time points. In total 92 UR samples and 77 UE samples were collected. The horses were defined as untrained because they had completed ≤ four sprint exercise bouts (e.g., work days) prior to sampling. The number of prior work days and days of submaximal prior training prior to sampling were recorded. Horses were defined as untrained in order to integrate results with those of Bryan et al. (2017), where the untrained cohort had performed only 1−2 work days prior to sampling, and the trained cohort had completed a mean of 15.1 work days prior to sampling (SD = 9.1).

### Exercise Test

The exercise stimulus was an intense sprint bout of exercise (work day) undertaken as part of normal training. The training regime for horses is submaximal training at canter six times per week, with work days being introduced and replacing one to two submaximal bouts per week. On a work day horses were initially walked on an automated horse walker for 30–60 min, followed by 5–10 min of walking in hand. Under saddle there was an initial warm-up period of 300 m walk and 700 m of trot and slow canter down the incline of the track. The work day was performed on a 1,500 m all-weather woodchip gallop track, with the final 800 m straight set on a 2.7% incline. The sprint portion of the exercise bout consisted of the horses galloping at high intensity for 800-1,000 m up the incline of the gallop. In a larger cohort of horses (*n* = 294) from the same training establishment, the work day was characterised using concurrent global positioning system (GPS) and heart rate monitoring (Farries et al., 2019). From 2,900 GPS recordings the mean peak speed was 16.36 m/s (range: 14.23−17.63 m/s). Of these 2,900 recordings 1,056 had simultaneous heart rate recordings, with a mean peak heart rate of 219 beats per minute (range: 182−237).

For 34/77 UE horses, whole blood was collected at rest and five minutes post-exercise into fluoride oxalate tubes. Samples were centrifuged, and plasma lactate concentrations measured on-site using a YSI2300 STAT PLUS auto analyser (YSI UK Ltd, Hampshire, UK). These measurements were used to validate the intensity of the exercise test performed.

### Biopsy Sampling

Percutaneous needle muscle biopsies (approximately 300 mg) were obtained from the ventral compartment of the middle gluteal muscle using the method described by Valette et al. (1999). All UR samples were collected between 7:30 am and 11:30 am. UE samples were taken four hours after completion of the exercise test, as this has previously been shown to be a timepoint where the greatest change in gene expression in response to acute exercise was observed (McGivney et al., 2009; Eivers et al., 2010). Muscle samples were stored in RNAlater (Thermo Fisher, Massachusetts, USA) for 24 hours at 4°C then stored at −20°C prior to RNA extraction.

### RNA Extraction and Quality Control

Total RNA was extracted from approximately 70 mg tissue using a protocol combining TRIzol reagent (Thermo Fisher), DNase I treatment (Qiagen, Hilden, Germany) and an RNeasy Mini-Kit (Qiagen). RNA was quantified using a Nano Drop ND1000 spectrophotometer V 3.5.27 (Thermo Fisher). RNA quality was assessed using the RNA integrity number (RIN) on an Agilent Bioanalyser with the RNA 6000 Nano LabChip kit6 (Agilent, Cork, Ireland).

### RNA Sequencing

Indexed, strand-specific Illumina sequencing libraries were prepared using the TruSeq Stranded mRNA Library Preparation Kit LT (Illumina, San Diego, CA, USA). Libraries were pooled with 18–20 indexed libraries per pool and sequenced on an Illumina HiSeq 2500 using a Rapid Run flow cell and reagents to generate 100 bp paired-end reads. Each pool was sequenced across both lanes of the flow cell (dual lane loading). Demultiplexed sequence data was then converted to FASTQ format. Sequencing was performed by the Research Technology Support Facility, Michigan State University.

### RNA-Seq Data Workflow

Quality control of the sequence reads was performed using *FastQC* [version: 0.11.5] (Andrews, 2010). *STAR* aligner [version: 2.5.2b] (Dobin et al., 2013) was used to map reads to the Equine reference genome EquCab2 (Ensembl release 62). After mapping, *featureCounts* [version: 1.5.0] was used to assign reads to genes (Liao et al., 2014). Data for each sample from each sequencing lane was then merged where concordance was >99% between lanes. Count data was parsed using a custom script, then small non-coding RNA were filtered using *BiomaRt* (Durinck et al., 2009). Assessment of the count data and multidimensional scaling were performed using *edgeR* (Robinson et al., 2010). Results of the multidimensional scaling were visualised using *ggplot2* (Wickham, 2009). Count data was quantile normalised using *preprocessCore* [version: 1.40.0] (Bolstad, 2017) within the R environment [version: 3.5.1] (R Core Team, 2017), and the log_2_ of quantile-normalised count data calculated.

### Genotyping

Genomic DNA was extracted from whole blood using the Maxwell 16 automated DNA purification system (Promega, Madison, WI, USA). Horses were genotyped on the Illumina Equine SNP70 BeadChip (Illumina). A genetic versus phenotypic sex check was performed. SNPs with a genotyping rate of <95%, and individuals with a genotyping rate <95% were excluded. SNPs with a minor allele frequency (MAF) < 0.10 were removed. Using these quality-controlled SNPs, identity by state (IBS) distances between individuals were calculated using the ‘genome’ function in *PLINK* [version 1.09] (Chang et al., 2015). The remaining 43,988 SNPs were then pruned based on pairwise linkage disequilibrium (LD) using a sliding window with an LD threshold of *r* *^2^* > 0.7, a window size of 50, and a step of 5 in *PLINK*. A set of 15,995 SNPs were used for the eQTL analysis. Pruning was undertaken due to the large spanning of LD within the Thoroughbred, with previous work validating the use of <15,000 SNPs to capture the majority of genetic variation (Corbin et al., 2014; Schaefer et al., 2017).

### eQTL Analysis

eQTL were determined using a linear model within *matrixEQTL* [version: 2.1.1] (Shabalin, 2012); including sex and age at sampling (days) as covariates. As samples had been included in two separate sample pools which were sequenced separately, the sequencing batch for each sample was also included as a covariate. Tests of association were corrected using the Benjamini-Hochberg procedure (Benjamini and Hochberg, 1995) and eQTL with a corrected *P*-value (*P*_FDR_) < 0.05 were catalogued for UR and UE samples separately. eQTL located within 1 Mb of the transcription start site (TSS) of the gene they were associated were designated as *cis*, and those located >1Mb from the TSS were designated *trans*, in keeping with human eQTL studies (Lonsdale et al., 2013). Significant results were then compared against genes previously identified in the skeletal muscle transcriptional response to acute, high-intensity exercise (a work day; 3,241 genes) and transcriptional response to a six-month period of training (3,405 genes) (Bryan et al., 2017).

### Functional Enrichment Analysis

Genes with significant eQTL were investigated for enrichment of biological processes using gene ontology (GO) categories (Ashburner et al., 2000) with the *clusterProfiler* package [version: 3.10.1] (Yu et al., 2012) within the R environment. Equine Ensembl IDs were mapped to annotated human orthologs, retrieved from the BioMart database (Kasprzyk, 2011) and GO enrichment performed using the annotation from the human genome annotation package *org.Hs.eg.db* [version: 2.12.0] (Carlson, 2019). The background gene set was the complement of genes expressed in skeletal muscle identified in this study (13,384 genes; 12,707 mapped to human orthologs). A threshold for significant enrichment was set at <0.05 after adjustment using the Benjamini-Hochberg procedure (*P*_FDR_) (Benjamini and Hochberg, 1995). The number of genes assigned to each Biological Process (Gene count) and proportion of genes associated with that cluster out of all the genes expressed (Gene ratio) were also reported. Results were visualised using the *clusterProfiler* package (Yu et al., 2012).

## Results

### Cohort

UR horses had a mean age of 611.7 days (range: 513–787 days), UE horses had a mean age of 757.5 days (range: 617–1,283 days). Dates of commencing preparatory training were available for 90 of the UR horses; 21 of the UR horses were sampled prior to breaking, 69 were sampled after breaking with a mean of 41.5 days after commencing preparatory training (range: 5-154 days) (**Table 1**). UE horses were sampled on average 156.6 days after commencing preparatory training (range: 31–307). UR horses had an average of 41.5 days submaximal training (range: 5–154) and UE had on average 48.6 (range: 19–152) (**Table 1**). UR horses had completed a mean of 0.3 work days (range: 0–4), UE horses completed a mean of 0.5 WDs prior to sampling (range: 0–3) (**Table 2**). A subset of 34 of the UE horses had a mean peak post-exercise plasma lactate concentration of 28.2 mmol/L, and a mean resting plasma concentration of 0.4 mmol/L. All RNA samples used for RNA-seq had a RIN greater than 7.0, the UR cohort had a mean RIN of 8.0 (range: 7.2−9.3) and the UE cohort had a mean RIN of 8.1 (range: 7.0−9.3). Multi-dimensional scaling was used to visually inspect the count data, showing separation of untrained resting and untrained post-exercise samples (**Figure S1**).

**Table 1:** Description of prior training completed by horses in the untrained resting and untrained post-exercise cohorts.

	Preparatory training		Submaximal training	
Untrained resting	No. horses sampled before entering preparatory training	21	No. horses sampled before entering submaximal training	52
	No. horses sampled after entering preparatory training	69	No. horses sampled after entering submaximal training	16
	Days preparatory training prior to sampling	Mean: 31.82 SD: 40.78 Range: 5-154	Days submaximal training prior to sampling	Mean: 11.44 SD: 26.16 Range: 19-152
Untrained post-exercise	No. horses sampled before entering preparatory training	0	No. horses sampled before entering submaximal training	0
	No. horses sampled after entering preparatory training	74	No. horses sampled after entering submaximal training	76
	Days preparatory training prior to sampling	Mean: 156.61 SD: 63.91 Range: 31-307	Days submaximal training prior to sampling	Mean: 124.59 SD: 77.61 Range: 7-302

**Table 2:** Number of prior high-intensity sprint bouts (work days, WDs) completed prior to sampling for horses within untrained resting and untrained post-exercise cohorts.

Untrained resting		Untrained post-exercise	
Prior WDs	No. horses	Prior WDs	No. horses
0	78	0	47
1	7	1	24
2	5	2	4
3	2	3	1
4	1	4	0

Analysis of the genetic relatedness of the cohort showed the mean IBS distance between individuals was 0.69 and ranged from 0.64−0.85 (SD = 0.03). Of the 19 sires represented in the cohort, the top six sires in terms of number of progent represented had 39, 23, 14, 10, 6, and 4 progeny. There were two sires with two progeny and the remaining ten sires had one offspring each. There were 12 full siblings in the cohort and 34 half-siblings by dam.

### eQTL Discovery

**Figure 1:**
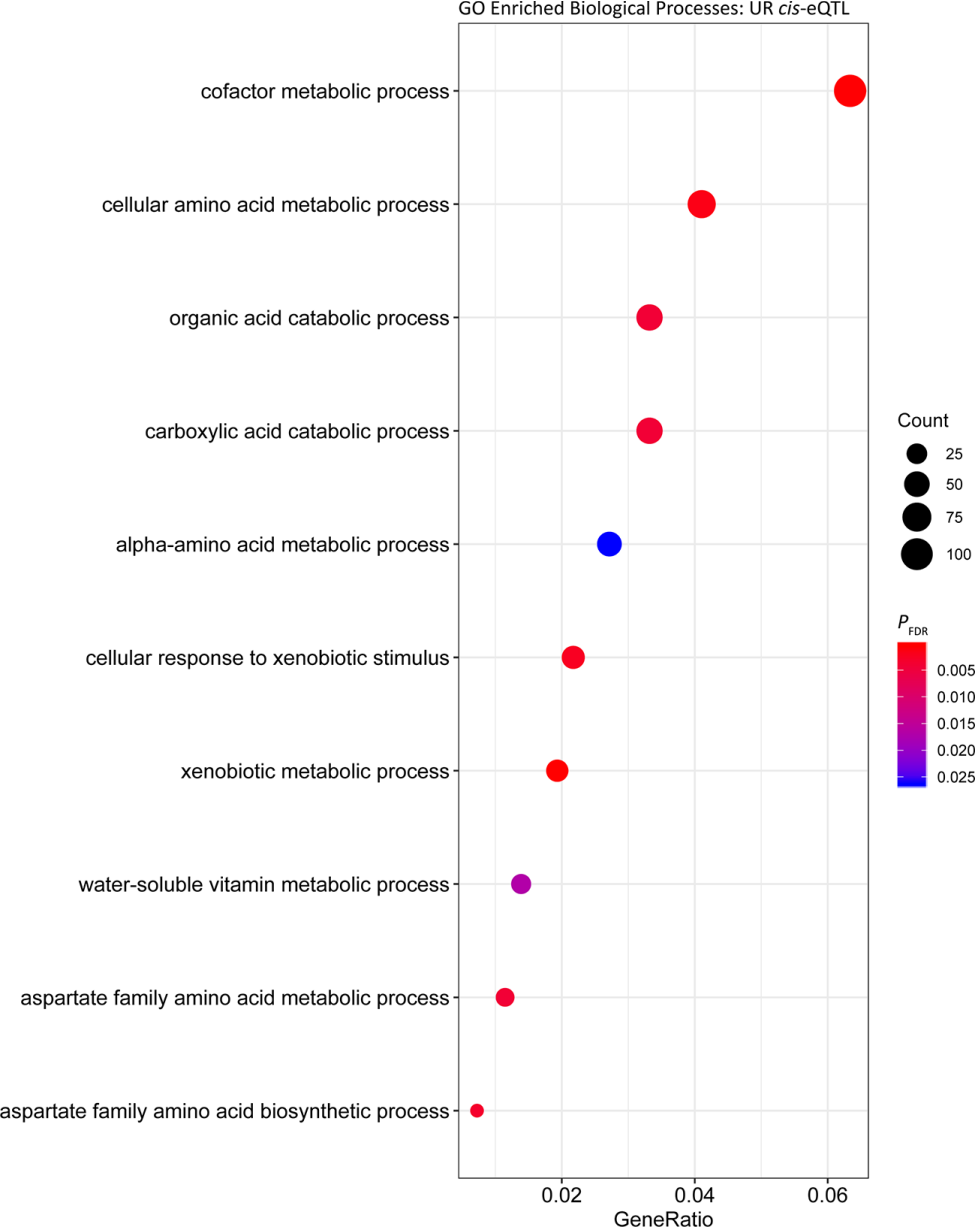
Gene ontology enrichment of biological processes for genes under *cis* regulation in the untrained resting cohort.

Using the full complement of 13,384 genes, 3,582 *cis*-eQTL and 1,703 *trans*-eQTL were detected in UR samples (*P*_FDR_ < 0.05). The 3,582 *cis*-eQTL were associated with expression of 1,456 genes. The gene with the strongest *cis*-eQTL (BIEC2-707785) in UR horses was the endosome associated trafficking regulator 1 gene (*ENTR1*; *P*_FDR_ = 3.81 × 10^-27^) (**Figure S2**, **Table 3**). GO enrichment analysis of the *cis* regulated genes in UR samples showed that the most significantly enriched Biological Process was ‘GO:0006805 xenobiotic metabolic process ‘ (*P*_FDR_ = 3.02 × 10^-7^, Gene Ratio = 33/1,614). ‘GO:0051186 cofactor metabolic process’ (*P* *_FDR_* = 1.42 × 10^-4^) was also significantly enriched and had the largest Gene Ratio (105/1,614) (**Figure 1**, **Table S1**).

**Table 3:** Top 10 genes by strongest *cis*-eQTL association.

SNP	Ensembl Gene ID	Gene	SNP Chr	SNP Pos	*P*_FDR_	Gene Chr	TSS	Gene End	Strand	Distance from TSS
**Untrained resting**										
BIEC2-707785	ENSECAG00000010463	*ENTR1*	25	37504251	3.81E-27	25	37555303	37560097	1	-51052
BIEC2-1078267	ENSECAG00000012661	NA	8	1998191	1.46E-24	8	1610405	1612946	-1	387786
BIEC2-1078267	ENSECAG00000021215	NA	8	1998191	3.61E-24	8	2897771	2900415	-1	-899580
BIEC2-1078267	ENSECAG00000020446	NA	8	1998191	5.51E-23	8	2440998	2443623	-1	-442807
BIEC2-1078267	ENSECAG00000019976	NA	8	1998191	3.23E-22	8	2341391	2343774	-1	-343200
BIEC2-737866	ENSECAG00000024388	*BPIFC*	28	30492661	3.20E-21	28	30663308	30696619	-1	-170647
BIEC2-1078267	ENSECAG00000014546	NA	8	1998191	5.14E-21	8	1868952	1871600	-1	129239
BIEC2-235541	ENSECAG00000019034	*NMRAL1*	13	38538682	2.28E-18	13	38492654	38500913	1	46028
BIEC2-312869	ENSECAG00000021118	NA	15	28568686	5.02E-18	15	29280622	29284093	-1	-711936
BIEC2-163619	ENSECAG00000003428	NA	11	58806041	8.51E-18	11	57863227	57863916	-1	942814
**Untrained post-exercise**										
BIEC2-707785	ENSECAG00000010463	*ENTR1*	25	37504251	1.66E-24	25	37555303	37560097	1	-51052
BIEC2-737866	ENSECAG00000024388	*BPIFC*	28	30492661	2.41E-21	28	30663308	30696619	-1	-170647
BIEC2-240006	ENSECAG00000017792	*PARN*	13	30544927	2.41E-21	13	30444515	30591937	1	100412
BIEC2-1078267	ENSECAG00000012661	NA	8	1998191	4.18E-18	8	1610405	1612946	-1	387786
BIEC2-741205	ENSECAG00000021492	*ADSL*	28	36754782	6.53E-18	28	37011649	37027102	1	-256867
BIEC2-328876	ENSECAG00000020459	*SEC13*	16	6720019	7.95E-18	16	6667915	6693504	1	52104
TBIEC2-918150	ENSECAG00000000861	*CHCHD3*	4	87261712	7.95E-18	4	86938001	87207374	-1	323711
BIEC2-166011	ENSECAG00000016949	NA	11	49589952	7.95E-18	11	49427265	49438385	1	162687
BIEC2-898764	ENSECAG00000013187	*LPGAT1*	5	26823295	3.92E-17	5	26734686	26804173	1	88609
BIEC2-62887	ENSECAG00000016304	*CASC4*	1	144947609	4.14E-17	1	1.45E+08	144833523	-1	220296

**Figure 2:**
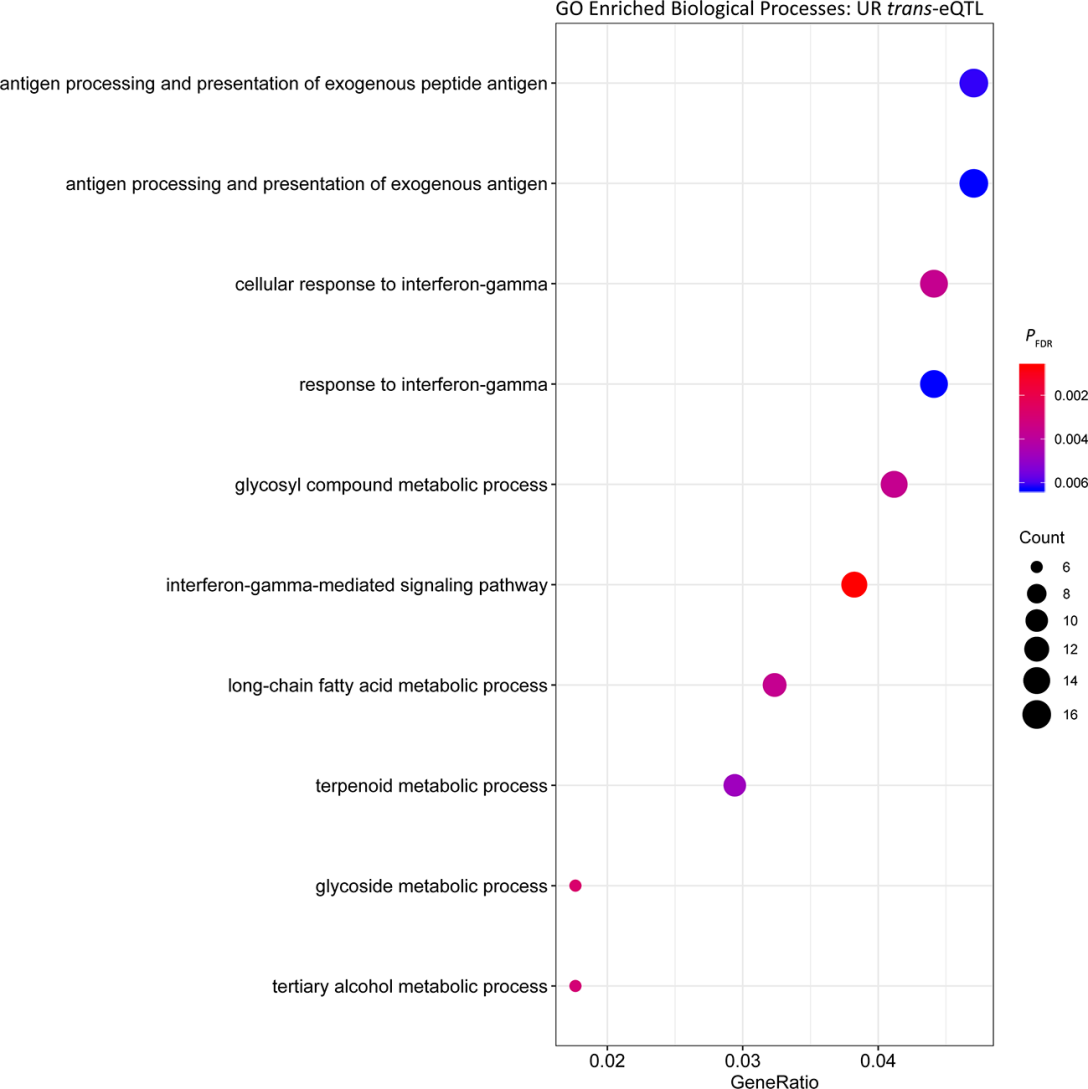
Gene ontology enrichment of biological processes for genes under *trans* regulation in the untrained resting cohort.

In the UR cohort 1,219 *trans*-eQTL were associated with 425 genes. The majority 70.39% (858) were located on the same chromosome as the associated gene, and 29.61% (361) were associated with genes located on different chromosomes. The most significant *trans*-eQTL was BIEC2-526896 on ECA20 and expression of the DEAH-box helicase 16 gene (*DHX16*) also located on ECA20 1.49 Mb downstream from BIEC2-526896 (*P*_FDR_ = 3.50 × 10^-17^) (**Table 4**). Functional analysis of the *trans* eGenes showed enrichment of ‘interferon-gamma-mediated signalling’ (*P*_FDR_ = 6.06 × 10^-4^, Gene Ratio = 13/340) (**Figure 2**, **Table S2**). The functional categories with the highest Gene Ratios were ‘cofactor metabolic process’ (*P* *_FDR_* = 0.01, Gene Ratio = 29/340) and ‘monocarboxylic acid metabolic process’ (*P* *_FDR_* = 0.02, Gene Ratio = 29/340) (**Figure 2**, **Table S2**).

**Table 4:** top ten genes by strongest *trans*-eQTL association.

SNP	Ensembl Gene ID	Gene	SNP Chr	SNP Pos	*P* *_FDR_*	Gene Chr	TSS	Gene End	Strand	Distance from TSS
**Untrained resting**										
BIEC2-526896	ENSECAG00000008537	*DHX16*	20	28206968	3.50E-17	20	29693915	29708818	-1	-1486947
BIEC2-218753	ENSECAG00000015585	*TBL2*	13	9657509	8.69E-14	13	11241845	11246395	-1	-1584336
BIEC2-526896	ENSECAG00000015782	NA	20	28206968	5.20E-13	20	29364710	29368260	1	-1157742
BIEC2-526896	ENSECAG00000015505	NA	20	28206968	3.57E-12	20	30173637	30178981	-1	-1966669
BIEC2-554291	ENSECAG00000019318	NA	20	29116874	3.57E-12	20	32450503	32452484	-1	-3333629
BIEC2-991035	ENSECAG00000012956	*DCPS*	7	31604226	9.10E-12	7	35117723	35153555	1	-3513497
BIEC2-526896	ENSECAG00000021750	NA	20	28206968	2.27E-11	20	31193941	31197235	-1	-2986973
BIEC2-526791	ENSECAG00000009368	NA	20	27782384	2.27E-11	20	29049395	29052907	1	-1267011
BIEC2-554291	ENSECAG00000021750	NA	20	29116874	4.38E-11	20	31193941	31197235	-1	-2077067
BIEC2-31048	ENSECAG00000024546	NA	1	73056328	5.71E-11	1	70638960	70656636	-1	2417368
**Untrained post-exercise**										
UKUL2765	ENSECAG00000010213	*MCCC2*	14	93893549	1.12E-13	14	92304084	92460487	-1	1589465
BIEC2-3702	ENSECAG00000004989	NA	1	9825107	4.38E-12	1	96257111	96258450	1	-86432004
BIEC2-991035	ENSECAG00000012956	*DCPS*	7	31604226	2.03E-11	7	35117723	35153555	1	-3513497
BIEC2-526896	ENSECAG00000015505	NA	20	28206968	6.37E-11	20	30173637	30178981	-1	-1966669
BIEC2-210079	ENSECAG00000012497	*LIMK1*	13	10604233	6.76E-11	13	11610902	11634978	1	-1006669
BIEC2-554291	ENSECAG00000019318	NA	20	29116874	2.91E-10	20	32450503	32452484	-1	-3333629
BIEC2-526896	ENSECAG00000015782	NA	20	28206968	4.06E-10	20	29364710	29368260	1	-1157742
BIEC2-554291	ENSECAG00000015505	NA	20	29116874	8.06E-10	20	30173637	30178981	-1	-1056763
BIEC2-63245	ENSECAG00000016304	*CASC4*	1	1.48E+08	1.04E-09	1	1.45E+08	1.45E+08	-1	3045045
BIEC2-3702	ENSECAG00000009972	*LHPP*	1	9825107	2.16E-09	1	8372229	8511110	-1	1452878

In the UE cohort 4,992 *cis*-eQTL were associated with the expression of 1,922 genes. The most significant *cis*-eQTL was BIEC2-707785 on ECA25 and *ENTR1* (*P*_FDR_ = 1.66 × 10^-24^) (**Figure S3**), as was the case in the UR cohort (**Table 3**). The strongest *trans*-eQTL association was between BIEC2-165011 on ECA11 and transcript ENSECAG00000016949 (*P*_FDR_ = 1.12 × 10^-13^) (**Table 4**). Similar to UR samples, the majority (75.45%; 544) of *trans*-eQTL were located on the same chromosome and 24.55% (177) were on different chromosomes.

**Figure 3:**
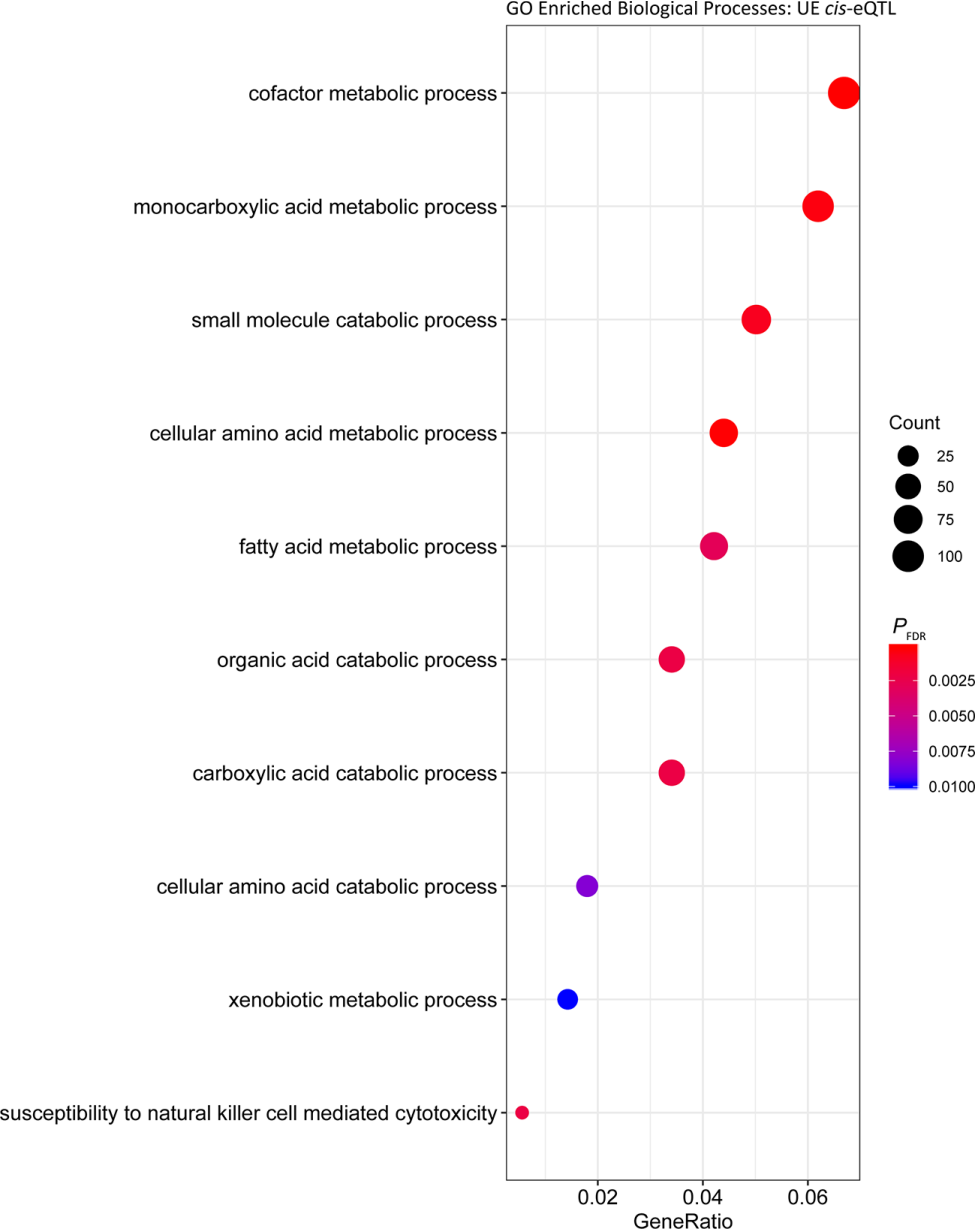
Gene ontology enrichment of biological processes for genes under *cis* regulation in the post-exercise cohort.

**Figure 4:**
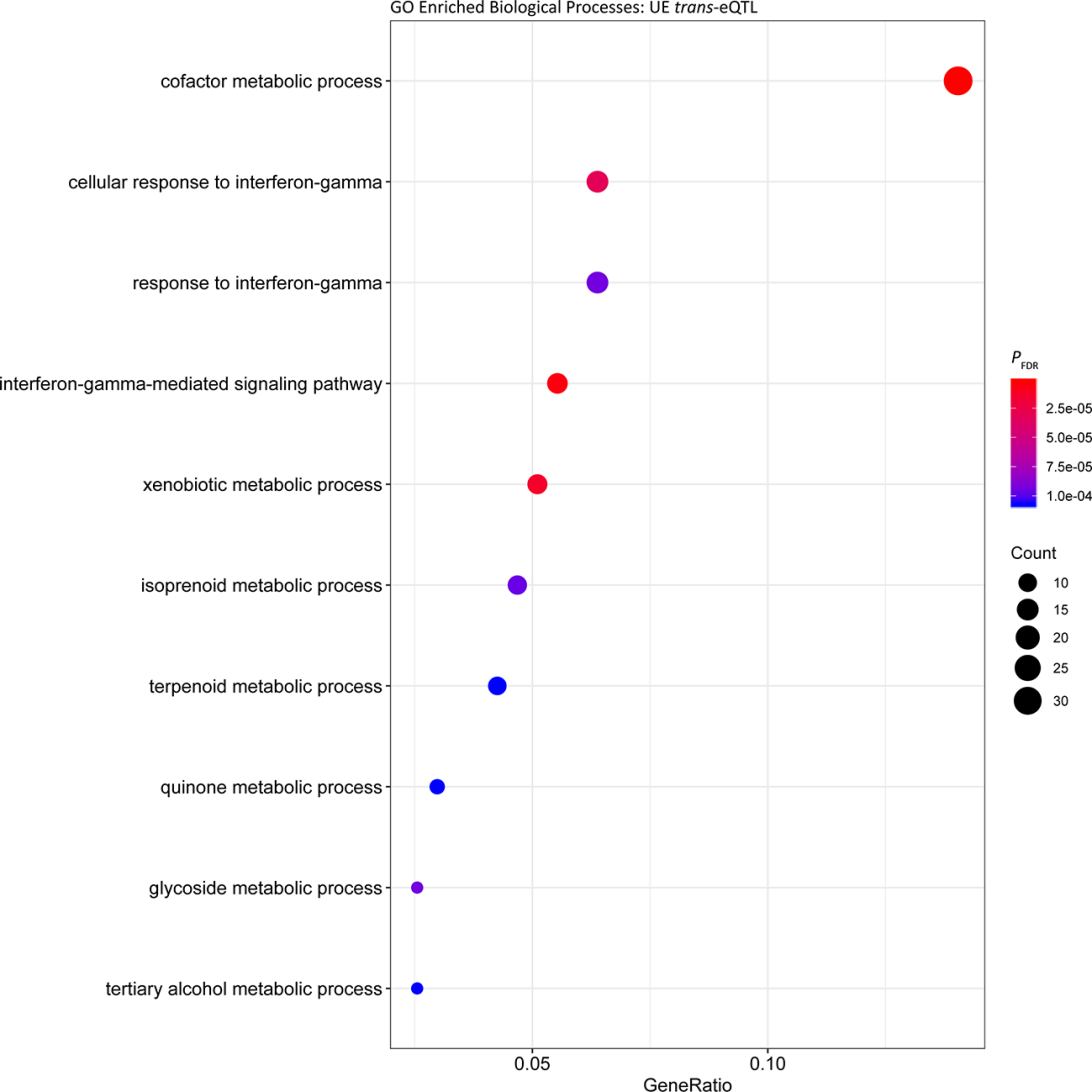
Gene ontology enrichment of biological processes for genes under *trans* regulation in the post-exercise cohort.

Analysis of the *cis* regulated genes in UE samples showed that similar to the UR cohort, the most significantly enriched Biological Process was ‘cofactor metabolic process’ (*P*_FDR_ = 6.40 × 10^-7^, Gene Ratio = 112/1,579) (**Figure 3**, **Table S3**). Comparable results were obtained for enrichment of Biological Processes among putative *trans* regulated genes, with ‘cofactor metabolic process’ the most significantly enriched (**Figure 4**; *P*_FDR_ = 5.06 × 10^-7^, Gene Ratio = 33/235).

### Genetic Regulation of Exercise Relevant Genes

The total set of genes with expression changes associated with eQTLs (i.e. eGenes) were queried against genes that we have reported from the same dataset to be differentially expressed post-exercise in a sample of 39 Thoroughbreds (Bryan et al., 2017). Of the 3,582 UR *cis*-eQTL, 913 were associated with genes differentially expressed in response to exercise. The most significant association was between BIEC2-285235 and the CCR4-NOT transcription complex subunit 11 gene (*CNOT11*; *P*_FDR_ = 3.00 × 10^-15^) (**Table 5**). Of the 1,703 UR *trans*-eQTL, 144 were associated with exercise relevant genes. The most significant *trans*-eQTL was between BIEC2-1061469 and the TAL bHLH transcription factor 2 gene (*TAL2*; *P*_FDR_ = 3.03 × 10^-10^) (**Table 6**).

**Table 5:** top ten *cis*-eQTL identified in genes differentially expressed in response to exercise.

SNP	Ensembl Gene ID	Gene	Log_2_FC: Exercise^1^	SNP Chr	SNP Pos	*P* *_FDR_*	Gene Chr	TSS	Gene End	Strand	Distance from TSS
**Untrained resting**											
BIEC2-285235	ENSECAG00000011967	*CNOT11*	0.34	15	8238549	3.00E-15	15	8266315	8276804	-1	-27766
BIEC2-240006	ENSECAG00000017792	*PARN*	0.37	13	30544927	4.42E-15	13	30444515	30591937	1	100412
BIEC2-209001	ENSECAG00000019190	*IFT22*	-0.36	13	9212034	5.33E-15	13	9228359	9233728	-1	-16325
BIEC2-1035081	ENSECAG00000008723	NA	0.47	7	14212942	5.82E-14	7	14574017	14585790	-1	-361075
BIEC2-830969	ENSECAG00000012898	*PNLDC1*	0.55	31	1778298	3.95E-13	31	2140239	2157442	1	-361941
BIEC2-872079	ENSECAG00000024368	*IPO9*	-0.62	30	28911273	1.23E-12	30	28818157	28849624	1	93116
BIEC2-988215	ENSECAG00000018541	*C2CD2L*	-0.59	7	26800468	4.48E-12	7	26815981	26823699	1	-15513
BIEC2-911228	ENSECAG00000024971	*GSTM4*	-0.45	5	57817054	4.38E-11	5	58455048	58459646	-1	-637994
BIEC2-609981	ENSECAG00000011021	*NDUFAF5*	0.41	22	9904659	7.88E-11	22	10479889	10512792	-1	-575230
TBIEC2-57788	ENSECAG00000013327	*SLC24A1*	0.82	1	1.27E+08	9.47E-11	1	126764822	1.27E+08	-1	-3157
**Untrained post-exercise**											
BIEC2-240006	ENSECAG00000017792	*PARN*	0.37	13	30544927	2.41E-21	13	30444515	30591937	1	100412
BIEC2-209001	ENSECAG00000019190	*IFT22*	-0.36	13	9212034	5.64E-13	13	9228359	9233728	-1	-16325
BIEC2-1082617	ENSECAG00000008479	*SDR16C5*	-0.43	9	27686807	3.39E-12	9	27293107	27307849	1	393700
BIEC2-1035081	ENSECAG00000008723	NA	0.47	7	14212942	6.06E-12	7	14574017	14585790	-1	-361075
TBIEC2-513760	ENSECAG00000000973	NA	-1.82	2	73037320	1.15E-11	2	72462318	72935365	1	575002
BIEC2-770455	ENSECAG00000011697	*TMCC3*	0.57	28	19829349	2.82E-11	28	19803131	19811873	-1	26218
BIEC2-14401	ENSECAG00000010786	*PGAM1*	0.48	1	32009184	6.25E-11	1	31823570	31825848	-1	185614
BIEC2-412279	ENSECAG00000023566	*DHRS9*	1.18	18	48009567	1.04E-10	18	48741376	48752360	1	-731809
BIEC2-1029754	ENSECAG00000019879	*ISCU*	0.50	8	11582493	2.78E-10	8	12361828	12395086	-1	-779335
BIEC2-909217	ENSECAG00000009353	*AGMO*	-0.47	4	48124305	3.15E-10	4	47940706	48126316	-1	183599

^1^Log_2_ fold-change (log_2_FC) of expression in response to exercise in Bryan et al., 2017.

**Table 6:** top ten *trans*-eQTL identified in genes differentially expressed in response to exercise.

SNP	Ensembl Gene ID	Gene	Log_2_FC: Exercise^1^	SNP Chr	SNP Pos	*P* *_FDR_*	Gene Chr	TSS	Gene End	Strand	Distance from TSS
**Untrained resting**											
BIEC2-658237	ENSECAG00000004995	*TAL2*	-1.67	25	9914109	3.03E-10	25	11725340	11725720	1	-1811231
BIEC2-53419	ENSECAG00000013327	*SLC24A1*	0.82	1	124846027	3.72E-08	1	126764822	126790389	-1	-1918795
BIEC2-58303	ENSECAG00000013327	*SLC24A1*	0.82	1	128180460	2.14E-06	1	126764822	126790389	-1	1415638
BIEC2-913863	ENSECAG00000024971	*GSTM4*	-0.45	5	63531114	5.08E-06	5	58455048	58459646	-1	5076066
BIEC2-959879	ENSECAG00000024971	*GSTM4*	-0.45	5	61035410	3.24E-05	5	58455048	58459646	-1	2580362
BIEC2-496328	ENSECAG00000014441	*ZNF330*	0.56	2	89041634	3.72E-05	2	90360628	90375365	-1	-1318994
BIEC2-311700	ENSECAG00000017760	*ELK3*	0.44	15	53973327	5.26E-05	28	21110553	21148433	1	NA
BIEC2-907346	ENSECAG00000018012	*SELENBP1*	-0.56	5	44309385	6.82E-05	5	45898879	45906904	1	-1589494
BIEC2-59128	ENSECAG00000013327	*SLC24A1*	0.82	1	129427658	1.09E-04	1	126764822	126790389	-1	2662836
BIEC2-60026	ENSECAG00000013327	*SLC24A1*	0.82	1	130739790	1.59E-04	1	126764822	126790389	-1	3974968
**Untrained post-exercise**											
BIEC2-1053404	ENSECAG00000011541	*PRDX2*	0.53	7	51698577	1.51E-08	7	45573319	45575352	1	6125258
BIEC2-996678	ENSECAG00000011541	*PRDX2*	0.53	7	41340513	2.58E-08	7	45573319	45575352	1	-4232806
BIEC2-658237	ENSECAG00000004995	*TAL2*	-1.67	25	9914109	9.79E-08	25	11725340	11725720	1	-1811231
BIEC2-1028898	ENSECAG00000019879	*ISCU*	0.50	8	10602506	1.29E-06	8	12361828	12395086	-1	-1759322
BIEC2-1028319	ENSECAG00000019879	*ISCU*	0.50	8	10149470	9.98E-06	8	12361828	12395086	-1	-2212358
BIEC2-1051774	ENSECAG00000023096	*TPGS2*	0.41	8	51698367	2.02E-05	8	56940922	56994571	-1	-5242555
BIEC2-420441	ENSECAG00000009547	*WDR12*	0.57	18	76611180	4.93E-05	18	77655352	77674750	-1	-1044172
TBIEC2-1144261	ENSECAG00000008479	*SDR16C5*	-0.43	9	30312051	4.94E-05	9	27293107	27307849	1	3018944
TBIEC2-1149509	ENSECAG00000008479	*SDR16C5*	-0.43	9	40285361	6.34E-05	9	27293107	27307849	1	12992254
BIEC2-1052388	ENSECAG00000023096	*TPGS2*	0.41	8	53636613	1.07E-04	8	56940922	56994571	-1	-3304309

^1^Log_2_ fold-change (log_2_FC) of expression in response to exercise in Bryan et al., 2017.

Within the UE cohort 4,992 *cis*-eQTL were identified, 1,132 of which were associated with genes differentially expressed post-exercise. The strongest association was between BIEC2-240006 and the polyA-specific ribonuclease gene (*PARN*; *P*_FDR_ = 2.41 × 10^-21^) (**Table 5**). Of the UR trans-eQTL, 121 eQTL were associated with eGenes in the transcriptional exercise response. The strongest *trans*-eQTL in the UE cohort was BIEC2-1053404, associated with expression of the peroxiredoxin 2 gene (*PRDX2*; *P*_FDR_ = 1.51 × 10^-8^) (**Table 6**).

### Genetic Regulation of Training Relevant Genes

Using 3,405 genes that were differentially expressed in response to training in a sample of 39 Thoroughbreds (Bryan et al, 2017), we examined our results based on eQTL associated with genes within this transcriptional response. Within the UR cohort, 609 of the 3,582 *cis*-eQTL were associated with training response genes. The strongest association was between BIEC2-1061469 and the spindle and expression of the kinetochore associated complex subunit 1 gene (*SKA1*; *P*_FDR_ = 9.80 × 10^-18^) (**Table 7**). Of the 1,703 UR *trans*-eQTL, 145 were associated with training response genes. The most significant association was between BIEC2-658237 and *TAL2* (*P*_FDR_ = 3.03 × 10^-10^) (**Table 8**).

**Table 7:** top *cis*-eQTL identified in genes differentially expressed in response to training.

SNP	Ensembl Gene ID	Gene	Log_2_FC: Training^1^	SNP Chr	SNP Pos	*P* *_FDR_*	Gene Chr	TSS	Gene End	Strand	Distance from TSS
**Untrained resting**											
BIEC2-1061469	ENSECAG00000023484	*SKA1*	0.41	8	69117228	9.80E-18	8	68814638	68828326	1	302590
BIEC2-1061072	ENSECAG00000023484	*SKA1*	0.41	8	68498709	3.57E-17	8	68814638	68828326	1	-315929
TBIEC2-1028240	ENSECAG00000022151	*MATK*	0.58	7	2020098	5.03E-13	7	2272120	2277087	-1	-252022
BIEC2-974177	ENSECAG00000022151	*MATK*	0.58	7	1546395	1.12E-12	7	2272120	2277087	-1	-725725
BIEC2-872079	ENSECAG00000024368	*IPO9*	-0.66	30	28911273	1.23E-12	30	28818157	28849624	1	93116
BIEC2-988215	ENSECAG00000018541	*C2CD2L*	-0.43	7	26800468	4.48E-12	7	26815981	26823699	1	-15513
BIEC2-472999	ENSECAG00000021368	*FGGY*	0.37	2	732213	7.94E-12	2	549904	834638	-1	182309
BIEC2-472598	ENSECAG00000015014	*ACADL*	0.44	2	248866	1.08E-11	2	124580	165039	-1	124286
BIEC2-609981	ENSECAG00000011021	*NDUFAF5*	0.43	22	9904659	7.88E-11	22	10479889	10512792	-1	-575230
TBIEC2-57788	ENSECAG00000013327	*SLC24A1*	0.74	1	1.27E+08	9.47E-11	1	1.27E+08	1.27E+08	-1	-3157
**Untrained post-exercise**											
UKUL3712	ENSECAG00000010475	*IL33*	0.74	23	27442828	8.07E-16	23	27442768	27454175	1	60
BIEC2-974177	ENSECAG00000022151	*MATK*	0.58	7	1546395	1.96E-14	7	2272120	2277087	-1	-725725
TBIEC2-1028240	ENSECAG00000022151	*MATK*	0.58	7	2020098	7.95E-14	7	2272120	2277087	-1	-252022
BIEC2-1082617	ENSECAG00000008479	*SDR16C5*	-0.33	9	27686807	3.39E-12	9	27293107	27307849	1	393700
BIEC2-472598	ENSECAG00000015014	*ACADL*	0.44	2	248866	8.29E-12	2	124580	165039	-1	124286
TBIEC2-513760	ENSECAG00000000973	NA	-0.85	2	73037320	1.15E-11	2	72462318	72935365	1	575002
BIEC2-360074	ENSECAG00000014129	*TMEM42*	0.34	16	42029863	2.23E-10	16	41642464	41643652	-1	387399
BIEC2-472999	ENSECAG00000021368	*FGGY*	0.37	2	732213	2.74E-10	2	549904	834638	-1	182309
BIEC2-1029754	ENSECAG00000019879	*ISCU*	0.44	8	11582493	2.78E-10	8	12361828	12395086	-1	-779335
BIEC2-136659	ENSECAG00000023268	*CALHM5*	0.54	10	64788579	3.77E-10	10	65017017	65021618	1	-228438

^1^Log_2_ fold-change (log_2_FC) of expression in response to training in Bryan et al., 2017.

**Table 8:** top *trans*-eQTL identified in genes differentially expressed in response to training.

SNP	Ensembl Gene ID	Gene	Log_2_FC: Training^1^	SNP Chr	SNP Pos	*P*_FDR_	Gene Chr	TSS	Gene End	Strand	Distance from TSS
**Untrained resting**											
BIEC2-658237	ENSECAG00000004995	*TAL2*	-1.63	25	9914109	3.03E-10	25	11725340	11725720	1	-1811231
BIEC2-1118794	ENSECAG00000023484	*SKA1*	0.41	8	67640813	4.93E-10	8	68814638	68828326	1	-1173825
BIEC2-53419	ENSECAG00000013327	*SLC24A1*	0.74	1	124846027	3.72E-08	1	126764822	126790389	-1	-1918795
BIEC2-58303	ENSECAG00000013327	*SLC24A1*	0.74	1	128180460	2.14E-06	1	126764822	126790389	-1	1415638
BIEC2-59128	ENSECAG00000013327	*SLC24A1*	0.74	1	129427658	1.09E-04	1	126764822	126790389	-1	2662836
BIEC2-196080	ENSECAG00000017776	*PITPNM1*	0.38	12	25661692	1.28E-04	12	27228025	27239903	-1	-1566333
BIEC2-542299	ENSECAG00000021368	*FGGY*	0.37	20	7321704	1.34E-04	2	549904	834638	-1	NA
BIEC2-60026	ENSECAG00000013327	*SLC24A1*	0.74	1	130739790	1.59E-04	1	126764822	126790389	-1	3974968
BIEC2-580450	ENSECAG00000011021	*NDUFAF5*	0.43	22	9444819	2.10E-04	22	10479889	10512792	-1	-1035070
BIEC2-328903	ENSECAG00000020701	*CAV3*	-0.37	16	6817902	2.43E-04	16	8001463	8011813	-1	-1183561
**Untrained post-exercise**											
BIEC2-1053404	ENSECAG00000011541	*PRDX2*	0.59	7	51698577	1.51E-08	7	45573319	45575352	1	6125258
BIEC2-996678	ENSECAG00000011541	*PRDX2*	0.59	7	41340513	2.58E-08	7	45573319	45575352	1	-4232806
BIEC2-658237	ENSECAG00000004995	*TAL2*	-1.63	25	9914109	9.79E-08	25	11725340	11725720	1	-1811231
BIEC2-1028898	ENSECAG00000019879	*ISCU*	0.45	8	10602506	1.29E-06	8	12361828	12395086	-1	-1759322
BIEC2-344350	ENSECAG00000014129	*TMEM42*	0.34	16	43032493	4.09E-06	16	41642464	41643652	-1	1390029
BIEC2-1028319	ENSECAG00000019879	*ISCU*	0.45	8	10149470	9.98E-06	8	12361828	12395086	-1	-2212358
BIEC2-130408	ENSECAG00000023268	*CALHM5*	0.54	10	67512852	2.28E-05	10	65017017	65021618	1	2495835
TBIEC2-1144261	ENSECAG00000008479	*SDR16C5*	-0.33	9	30312051	4.94E-05	9	27293107	27307849	1	3018944
BIEC2-809036	ENSECAG00000005431	*CLRN2*	0.67	3	107506494	6.34E-05	3	106028106	106037626	-1	1478388
TBIEC2-1149509	ENSECAG00000008479	*SDR16C5*	-0.33	9	40285361	6.34E-05	9	27293107	27307849	1	12992254

^1^Log_2_ fold-change (log_2_FC) of expression in response to training in Bryan et al., 2017.

Within the UE cohort 766 of the 4,992 *cis*-eQTL were associated with training response genes. The most significant *cis*-eQTL association was between UKUL3712 and the interleukin 33 gene (*IL33*; *P*_FDR_ = 8.07 × 10^-16^) (**Table 7**). Of the 1,219 UE *trans*-eQTL 90 were associated with genes relevant to training. As with the exercise relevant genes, the strongest UE *trans*-eQTL was between BIEC2-1053404 and *PRDX2* (*P*_FDR_ = 1.51 × 10^-8^) (**Table 8**).

## Discussion

Using a systems genetics approach we have integrated RNA-seq and genome-wide SNP data for a large cohort of Thoroughbred horses in active race training that were maintained in a single environment. This strategy has allowed us to detect significant *cis* and *trans* eQTL in equine skeletal muscle that are likely to be relevant to an exercise phenotype, adaptation to training, an important and valuable trait in the racing Thoroughbred. A total of 4,992 *cis*-eQTL associated with the expression of 1,922 distinct genes were identified in the UR cohort; and 4,886 *cis*-eQTL associated with the expression of 1,875 genes were identified in the UE cohort. Fewer *trans*-eQTL were detected (UR: 1,703; UE: 1,219), which is consistent with previous studies, and likely due to the greater statistical power required to identify *trans*-eQTL (Westra and Franke, 2014).

The gene with the most significant association with a *cis*-eQTL in the UR and UE cohorts was *ENTR1* (**Table 3**, UR: *P* *_FDR_* = 3.81 × 10^-27^, UE: *P* *_FDR_* = 1.66 × 10^-24^). The ENTR1 protein is involved in cellular transport of cargo proteins from the endosome to the Golgi apparatus or for degradation in the lysosome (McGough et al., 2014) and has been suggested to play a role in cytokinesis (Hagemann et al., 2013). In a study where ENTR1 protein expression was blocked by RNA interference, there was a decrease in solute carrier family 2 member 1 glucose transporter protein (SLC2A1; previously known as GLUT1) (McGough et al., 2014). When examining whether this was due to increased SLC2A1 degradation, there was no evidence of increased transport of SLC2A1 to the lysosome. It was hypothesised that the decrease in SLC2A1 was mediated through regulation of transcription by ENTR1. SLC2A1 is responsible for approximately 30−40% of the glucose uptake in skeletal muscle, with the remainder transported through GLUT4 (Zisman et al., 2000; Rudich et al., 2003). As opposed to GLUT4 which is primarily expressed in skeletal muscle, SLC2A1 is widely expressed and is highly expressed on erythrocyte membranes (Krook et al., 2004). The control of SLC2A1 by ENTR1 in the context of the equine athlete is intriguing to speculate since SLC2A1 is expressed within equine lamellar tissue, and its expression is increased in hyperinsulinemia, therefore may play a role in the pathophysiology of equine laminitis (Campolo et al., 2016). *SLC2A1* is also differentially expressed in response to hypoxia, this has also been shown in equine chondrocytes *in vitro* after exposure to cobalt chloride (to mimic hypoxia) and in chondrocytes from osteoarthritis cases (Peansukmanee et al., 2009).

The most significant *trans* association in the UR cohort was between BIEC2-526896 and expression of the DEAH-box helicase 16 gene (*DHX16*) (**Table 4**). *DHX16* is an RNA helicase and is involved in regulation of translation and pre-mRNA splicing (Gencheva et al., 2010; Putiri and Pelegri, 2011). The gene located closest to BIEC2-526896 is the olfactory receptor family 12 subfamily D member 3 gene (*OR12D3*) with the TSS located 96.5 kb from the SNP. However, the zinc finger protein 311 gene (ZNF311) also relatively close to BIEC2-526896 (792.5 kb)(Consortium, 2017). *ZNF311* has previously been associated with telomere length in heterozygous ataxia-talengiectasia mutated (*ATM*) gene patients (Renault et al., 2017). As a member of the a krueppel c2h2-type zinc-finger protein family it is likely a transcription factor and has been associated with Biological Processes such as ‘regulation of transcription, DNA templated’ and ‘regulation of transcription by RNA polymerase II’(Consortium, 2017). The *trans* association between BIEC2-526896 and *DHX16* expression may therefore be mediated *via* the gene regulatory function of *ZNF311*.

The most significant *trans*-eQTL in the UE cohort was UKUL2765 and expression of the methylcrotonoyl-CoA carboxylase 2 gene (*MCCC2*) (**Table 4**). *MCCC2* encodes a subunit of 3-methylcrotonyl-CoA carboxylase (MCC), an enzyme which catabolises leucine (Stadler et al., 2005). Mutations within *MCCC2* have been found to result in MCC deficiency, which has varying implications for patients from no symptoms at all to death in early infancy (Fonseca et al., 2016). To date studies have yet to discern mutations which result in more or less severe disease phenotypes (Gallardo et al., 2001; Stadler et al., 2006). In terms of muscle physiology, *MCCC2* has been shown to be highly expressed in skeletal muscle of the red seabream fish (*Pagrus major*), which is likely due to high levels of protein metabolism within skeletal muscle (Abe et al., 2004). The TSS of the jumonji domain containing 4 gene (*JMJD4*) is located 71 bp from UKUL2765. The JMJD4 protein catalyses the hydroxylation of translation termination factor eRF1 lysine 63, which in turn enables the correct termination of translation and maintenance of translational fidelity (Feng et al., 2014). It is possible that the variation proximal to *JMJD4* is influencing expression of *JMJD4*, in turn altering expression of *MCCC2*. However, from the data available only one significant *cis*-eQTL for *JMJD4* was detected in the UR cohort and this was BIEC2-277622 located 257.8 kb downstream of the TSS (*P*_FDR_ = 6.58 × 10^-5^). Therefore it is not clear if UKUL2765 is tagging variation influencing *JMJD4* expression and mediating its influence on *MCCC2* through *JMJD4*.

Examination of eGenes previously shown to be involved in the skeletal muscle transcriptional response to exercise and training demonstrated that *TAL2* exhibited the most significant *trans*-eQTL in the UR cohort (BIEC2-658237; **Table 6**) and that this *trans*-eQTL was also highly significant in the UE cohort (*P*_FDR_ = 9.80 × 10^-8^; **Table 4**). *TAL2* encodes a basic-helix-loop-helix transcription factor (Xia et al., 1991; Langlands et al., 1997). Deletion of *TAL2* in mice has been shown to cause severe disruption of the development of the central nervous system, with new-born mice dying shortly post-partum (Bucher et al., 2000). *TAL2* has been shown to be vital for the development of gamma-aminobutyric acid (GABA, inhibitory neurotransmitter) signalling neurons in the developing midbrain, showing highly regulated and coordinated expression (Achim et al., 2013). When expression of *TAL2* was inhibited, neurons more closely resembled an excitatory glutamatergic phenotype (Achim et al., 2013). In terms of application in racing performance, GABA has previously been used as a calming agent in Thoroughbred racehorses, although it was banned from use in 2012. The GABA type A receptor associated protein like 1 gene (*GABARAPL1*) was also identified as a key regulator in the skeletal muscle transcriptional response to exercise (Bryan et al., 2017). In addition, we have previously reported functional enrichment of pathways related to neurodegenerative disorders in the transcriptional response to exercise (Bryan et al., 2017). Given the role of *TAL2* in GABAergic neuronal fate, this suggests a potential role for *TAL2* in the coordination of the response to exercise. These results suggest that the role of genes associated with neuronal differentiation and disease in the context of muscle and exercise warrants further investigation.

To identify common biological functions within genes identified under *cis* or *trans* regulation, enrichment analysis of Biological Processes among the gene sets was performed. Among the *cis* eGenes detected in both the UR and UE cohort, as well as *trans* eGenes in the UR cohort there was significant enrichment of cofactor metabolic processes (GO:0051186, **Tables S1**, **S3**, and **S4**). Cofactor metabolic process is defined as chemical reactions and pathways requiring the activity of an inorganic cofactor, such as an ion, or an organic coenzyme for the activity of an enzyme or other functional protein. Genes within this cluster were related to metabolism and substrate utilisation, including vitamin and mineral binding and synthesis such as: selenium (selenium binding protein 1 gene, *SELENBP1*; and selenoprotein T gene, *SELENOT*), molybdenum (molybdenum cofactor sulfurase gene, *MOCOS*) and thiamine (thiamine triphosphatase gene, *THTPA*). Consequently, variation in the expression of genes associated with nutrient binding may lead to variation in the ability of horses to utilise such nutrients. In this regard, abundance of selenoprotein gene transcripts has been used to identify dietary requirements for selenium in rats and turkeys (Barnes et al., 2009; Taylor and Sunde, 2017). Given the inter-animal variation in expression observed for genes relevant to substrate binding, it may be possible to use this information to evaluate nutrient requirements for individual horses, or whether expression of these genes can be modulated through diet.

Many of these genes have also been shown to have functions relevant to exercise, and variation within the expression of these genes may underpin variation in athletic performance. For example, the selenium binding protein 1 gene (*SELENBP1*) is significantly downregulated in response to exercise (log_2_FC = −0.56; *P*_FDR_ = 3.71 × 10^-11^) (Bryan et al., 2017). In both normal and cancerous human cells *SELENBP1* has been shown to be highly variable in expression (Yang and Sytkowski, 1998). Functionally, the SELENBP1 protein has been shown to be involved in many cellular processes including detoxification (Ishii et al., 1996), cytoskeletal outgrowth (Miyaguchi, 2004) and regulation of reduction and oxidation within the cell (Jamba et al., 1997). *SELENBP1* was found be differentially expressed in blood in response to administration of human recombinant erythropoietin in human endurance athletes (Durussel et al., 2016; Wang et al., 2017), suggesting a potential role in haematopoiesis and its regulation. In the UE cohort; the DDB1 and CUL4 associated factor 12 (*DCAF12*) and guanosine monophosphate reductase (*GMPR*) genes both exhibited significant *cis*-eQTL (*DCAF12*: *P*_FDR_ = 0.02; *GMPR*: *P*_FDR_ = 4.17 × 10^-3^). These genes, in addition to *SELENBP1*, were also shown by Wang et al. (2017) to be differentially expressed in blood in response to human recombinant erythropoietin. Variation in the expression of these genes may therefore potentially underpin variation in haematological phenotypes in horses, which may in turn influence traits relevant to aerobic capacity. It is also noteworthy that selenium deficiency has been associated with significant myopathy (White muscle disease) (Lofstedt, 1997; Delesalle et al., 2017) and reduced exercise tolerance in horses (Brady et al., 1978; Avellini et al., 1999). In addition, selenoproteins have been shown to be involved in several metabolic pathways and the response to oxidative stress in muscle (Rederstorff et al., 2006). These findings suggest an important role for selenium, and its associated biochemical machinery, in the correct functioning of skeletal muscle and muscle metabolism. This highlights the importance of selenium in the context of exercise and provides a potential role for variation in expression of genes relevant to selenium metabolism in determining metabolic function within the muscle.

The cofactor metabolic process cluster also contained genes relevant to mitochondrial function and oxidative phosphorylation. These included genes within the coenzyme Q synthesis pathway: coenzyme Q3 hydroxylase (*COQ3*), coenzyme Q7 (*COQ7*) and coenzyme Q8A (*COQ8A*). The coenzyme Q complex is a critical component of the electron transport chain during oxidative phosphorylation, moving electrons from complexes I and II to complex III (Lenaz, 1985; Turunen et al., 2004; Stefely and Pagliarini, 2017). COQ7 and COQ8A are required for coenzyme Q biosynthesis (Mollet et al., 2008; Stefely et al., 2016). Human patients with *COQ8A* mutations suffered seizures and other neurological symptoms and showed reduced coenzyme Q within skeletal muscle (Jacobsen et al.; Mollet et al., 2008). An eQTL for *COQ8A* in skeletal muscle has already been identified in horses, with a 227 bp SINE insertion in the promotor region of *MSTN* (g.66495326_66495327ins227) on ECA18 associated with increased expression of *COQ8A* (previously known as *ADCK3*) in Thoroughbreds (Rooney et al., 2017). However it should be noted that this increase in COQ8A expression did not appear to accompany an COQ8A protein abundance, with no difference in COQ8A protein abundance across genotypes (Rooney et al., 2017). This may be due to COQ8A having a regulatory role in coenzyme Q biosynthesis (Acosta et al., 2016). Electron transport chain complex activity assays, as well as assays using the exogenous application of ubiquinone, suggested a difference in the abundance of coenzyme Q across genotypes at this locus (Rooney et al., 2017). Suggesting variation at this SINE insertion is associated with *COQ8A* expression as well as coenzyme Q abundance. Therefore eQTL in the current study associated with *COQ8A*(**Figure S4**), and indeed other genes within the coenzyme Q biosynthetic pathway, may result in variation in synthesis of the coenzyme Q complex and have downstream implications for mitochondrial function.

We have for the first time systematically catalogued eQTL in equine skeletal muscle, both at rest and post-exercise. Previous investigations of eQTL in skeletal muscle have focussed primarily on human T2D (Sharma et al., 2011; Keildson et al., 2014a; Sajuthi et al., 2016; Langefeld et al., 2018) and meat quality traits in production animals (Ponsuksili et al., 2015; Gonzalez-Prendes et al., 2017; Pampouille et al., 2018; Gonzalez-Prendes et al., 2019; Velez-Irizarry et al., 2019). Our investigation of eQTL in the context of skeletal muscle and exercise present some of the only work to-date in this area (Kelly et al., 2014). Our work utilised linear models to detect associations between SNPs and gene expression as quantified by RNA-seq. It should be noted at this point that there are potential biases introduced in terms of the high number of related individuals within the cohort, future work could utilise more sophisticated techniques such as allele specific expression, where transcripts are mapped back to the maternal and paternal chromosomes and the expression of the maternal and paternal transcripts can be compared (Chamberlain et al., 2015). This would be particularly useful in our cohort given the high number of offspring by a small number of sires. Within the cohort there is also some variation in the amount of training prior to sampling, this was kept to a minimum by our sampling criteria. However, extending the study to incorporate trained horses and utilise variation in prior training by modelling the transcriptional training response could provide information on regulation of the training responsive transcriptome.

Our current results provide novel information concerning the regulation of gene expression in horses and can provide a framework for interpreting future GWAS of athletic and performance traits in Thoroughbreds. In terms of future applications of these results, the identification of quantitative trait transcripts (QTT) for athletic traits characterised in our cohort could be used to detect associations between a SNP, variation in expression of a QTT and a trait of interest. Thus giving a fuller picture of genetic variation contributing to traits of interest. An example may be detecting loci and QTT involved in the response to exercise and training, which has previously been shown to be highly heritable in humans (Timmons et al., 2010; Bouchard et al., 2011). The use of systems genetics approaches that integrate differential gene expression with genome variation represent an excellent strategy for dissecting the genetic architecture of complex anatomical and physiological traits.

## Data Availability Statement

The RNA-seq datasets analyzed for this study can be found in EBI ArrayExpress https://www.ebi.ac.uk/arrayexpress/experiments/E-MTAB-5447/. SNP datasets will not be made publicly available as the data were generated from privately owned horses, with a legal commitment to confidentiality. Researchers may request access to the data and consideration will be given to individuals following the conclusion of a confidentiality agreement. Requests should be made to the UCD Technology Transfer Office (https://www.ucd.ie/innovation/knowledge-transfer/).

## Ethics Statement

University College Dublin Animal Research Ethics Committee approval (AREC-P-12-55-Hill), a licence from the Department of Health (B100/3525) and informed owner consent were obtained.

## Author Contributions

GF performed computations and functional analyses. KB and PM assisted in analysis and pipeline development. CM, KG and GF performed biopsy sample collections. JB and GF prepared RNA. GF wrote the manuscript in close consultation with EH, LK and DM. All authors were involved in study design, implementation of the research and preparation of the manuscript.

## Funding

This research was funded by Science Foundation Ireland (SFI/11/PI/1166 and 18/TIDA/6019).

## Conflict of Interest

EH was employed by Plusvital Ltd. Plusvital Ltd had no role, financial or otherwise, in the research.EH, DM and LK are named inventors on multiple international patents relating to the application of variation in the prediction of race distance performance; none of which is relevant to the data/results reported in this manuscript.

The remaining authors declare that the research was conducted in the absence of any commercial or financial relationships that could be construed as a potential conflict of interest.

## Supplementary Material

Figure S1 Principal component analysis of quantile-normalised log counts of RNA-seq transcripts and coloured by sample type: untrained resting or untrained post-exercise.

Figure S2 Boxplot of ENTR1 expression (log2 quantile-normalised counts) across BIEC2-707785 genotypes in untrained resting samples.

Figure S3 Boxplot of ENTR1 expression (log2 quantile-normalised counts) across BIEC2-707785 genotypes in untrained post-exercise samples.

Figure S4 Boxplot of COQ8A expression (log2 quantile-normalised counts) across BIEC2-417075 genotypes in untrained post-exercise samples.
