# Supplementary figures and images for: Expression Quantitative Trait Loci in Equine Skeletal Muscle Reveals Heritable Variation in Metabolism and the Training Responsive Transcriptome
Source: Front Genet. 2019 Nov 26;10:1215. doi: 10.3389/fgene.2019.01215 (PMC6902038; doi:10.3389/fgene.2019.01215)

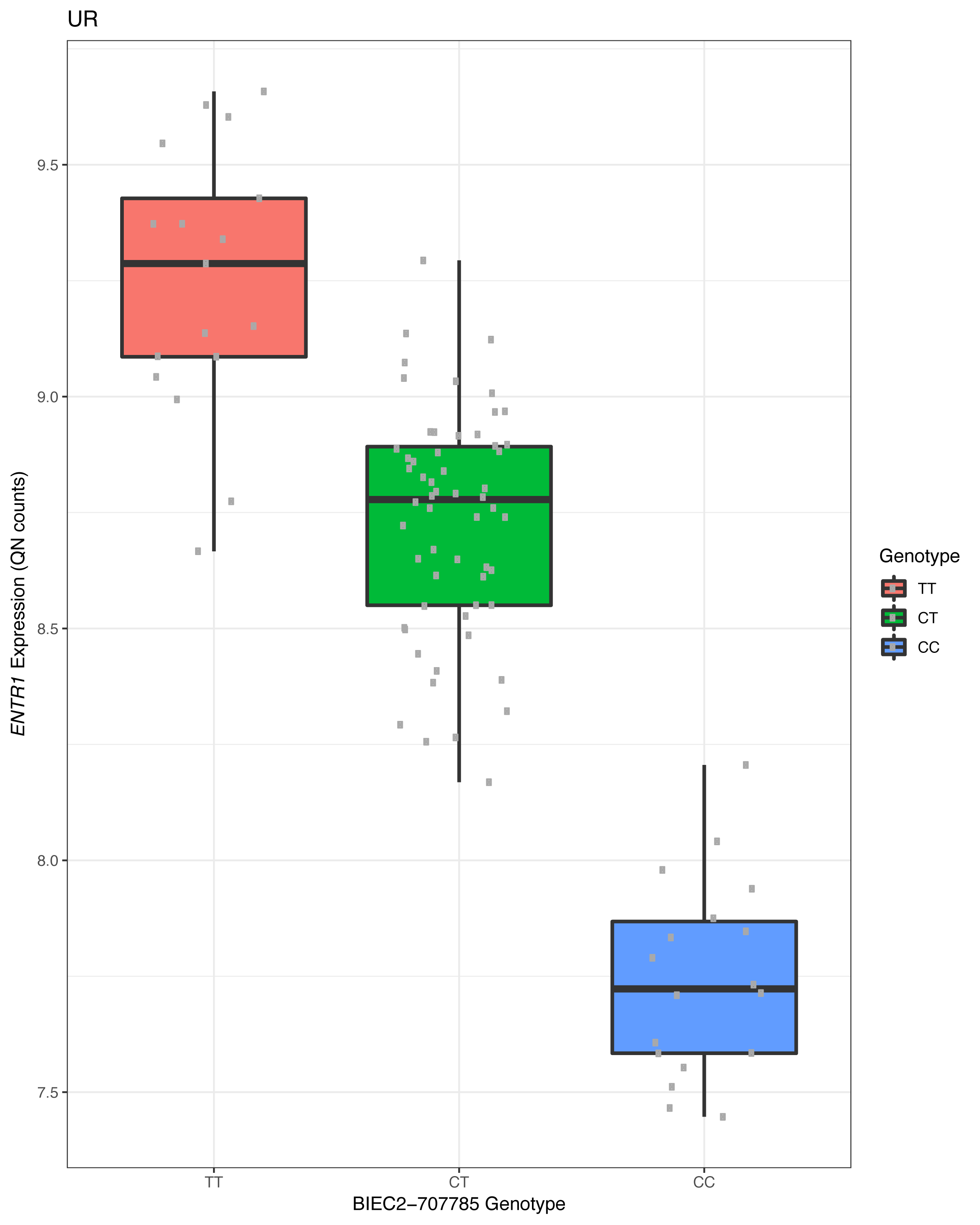

Supplement: Figure S1 — Principal component analysis of quantile-normalised log counts of RNA-seq transcripts and coloured by sample type: untrained resting or untrained post-exercise. [file Image_1.tif]

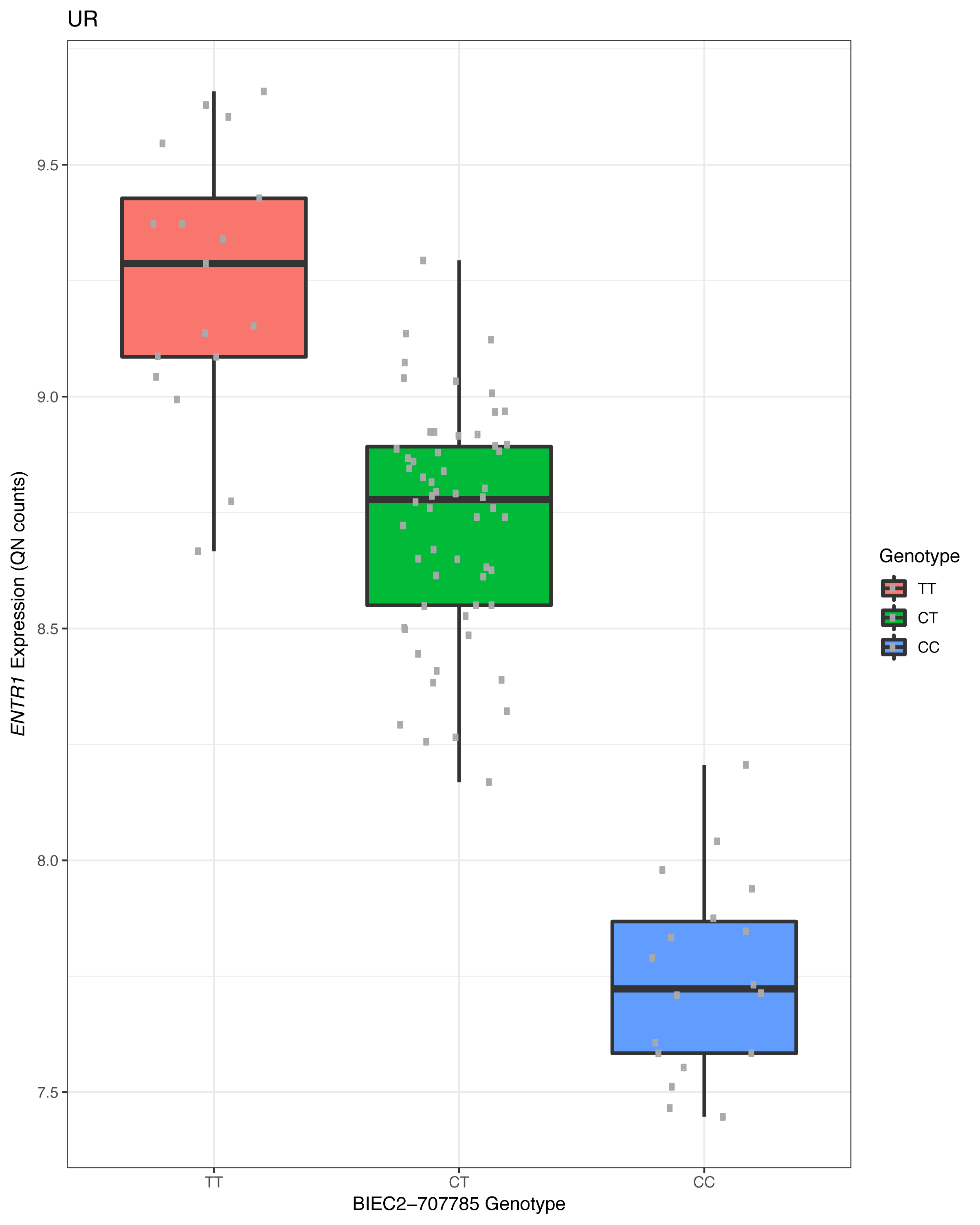

Supplement: Figure S2 — Boxplot of ENTR1 expression (log2 quantile-normalised counts) across BIEC2-707785 genotypes in untrained resting samples. [file Image_2.tif]

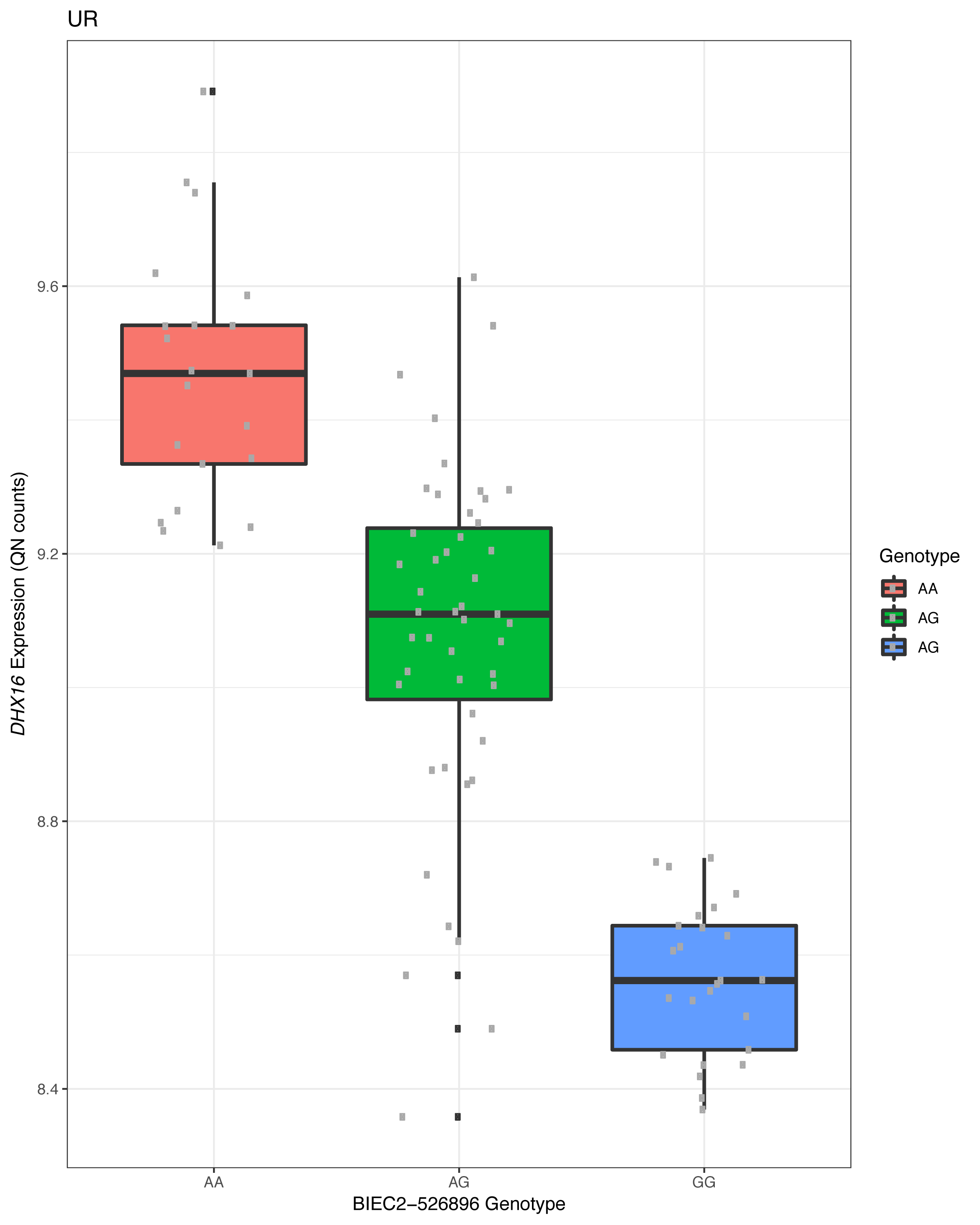

Supplement: Figure S3 — Boxplot of ENTR1 expression (log2 quantile-normalised counts) across BIEC2-707785 genotypes in untrained post-exercise samples. [file Image_3.tif]

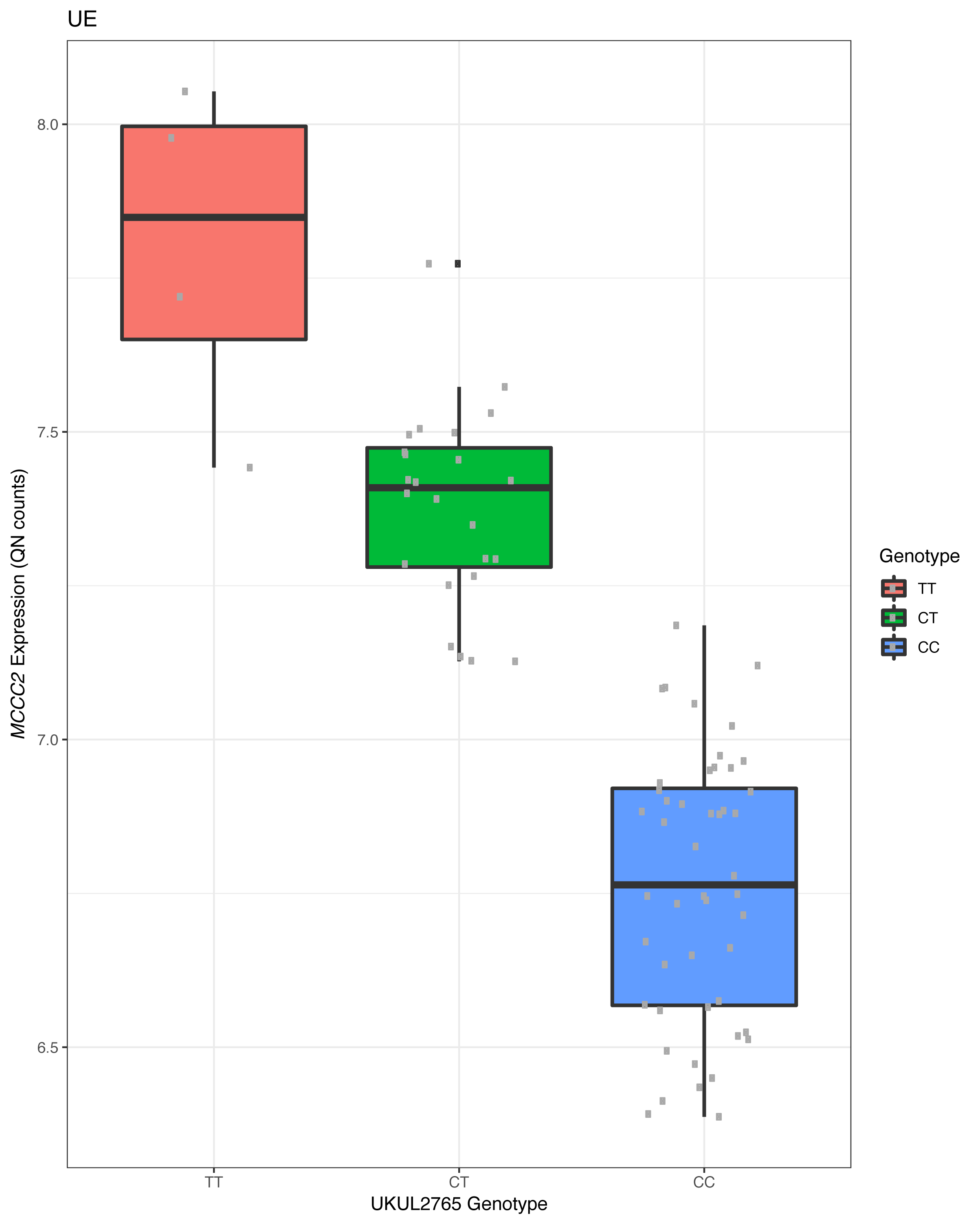

Supplement: Figure S4 — Boxplot of COQ8A expression (log2 quantile-normalised counts) across BIEC2-417075 genotypes in untrained post-exercise samples. [file Image_4.tif]
